# Nucleic Acid-based Detection of Bacterial Pathogens Using Integrated Microfluidic Platform Systems

**DOI:** 10.3390/s90503713

**Published:** 2009-05-18

**Authors:** Clarissa Lui, Nathaniel C. Cady, Carl A. Batt

**Affiliations:** 1 Department of Biomedical Engineering / Cornell University, 317 Stocking Hall, Ithaca, NY 14853, USA; 2 College of Nanoscale Science and Engineering / University at Albany State University of New York, 255 Fuller Rd., Albany, NY 12203, USA; E-Mail: ncady@uamail.albany.edu (N.C.C.); 3 Department of Food Science / Cornell University, 312 Stocking Hall, Ithaca, NY 14853, USA; E-Mail: cab10@cornell.edu (C.A.B.)

**Keywords:** pathogen, sensors, biosensors, PCR, DNA, microfluidics, μTAS, lab-on-a-chip

## Abstract

The advent of nucleic acid-based pathogen detection methods offers increased sensitivity and specificity over traditional microbiological techniques, driving the development of portable, integrated biosensors. The miniaturization and automation of integrated detection systems presents a significant advantage for rapid, portable field-based testing. In this review, we highlight current developments and directions in nucleic acid-based micro total analysis systems for the detection of bacterial pathogens. Recent progress in the miniaturization of microfluidic processing steps for cell capture, DNA extraction and purification, polymerase chain reaction, and product detection are detailed. Discussions include strategies and challenges for implementation of an integrated portable platform.

## 1. Introduction

The rapid, reliable detection of pathogenic bacteria is imperative in many different industries, of which food and agriculture, healthcare, environmental monitoring, and bio-defense are the four main players [1]. With recent devastating outbreaks of *Salmonella* and *Escherichia coli* in the United States, the food industry is largely concerned with the detection of pathogenic bacteria in agricultural products and processed foods. The presence of pathogenic bacteria can cost the food industry and consumers many millions of dollars every year due to food recalls, and is estimated to cause over 30,000 hospitalizations and over 1,000 deaths each year in the United States [2]. In the healthcare sector, approximately 25% of physician visits are caused by infectious diseases, many due to pathogenic agents. The ongoing evolution of microbes due to changing ecological, environmental, and human demographical factors necessitates improvements in the readiness of healthy and emergency service providers to respond to threats through effective surveillance, treatment, and control measures [3]. The development of a fast and sensitive platform for the detection of pathogens in human blood and waste samples is required in order to implement a quick and effective response to an outbreak. In the environmental monitoring arena, considerable attention is given to the evaluation of microbial cells in water and environment quality control, as well as for the study of microorganisms evolution and populations, for example in bio-waste composting substrates and their communities [4]. In the area of biodefense, biological agents are considered far more difficult to detect and defend against than chemical agents, and with bioterrorism now an issue of serious concern, the technology to counter a potential incident needs to be in place. To date, a multitude of reviews on micro total analysis systems for nucleic acid-based detection and microchip pathogen detection methods have been published [1,2,5-19], demonstrating great interest in the development of this field. A comprehensive literature survey was carried out for this present paper, and due to the immense amount of literature related to pathogenic detection, our study focuses primarily on rapid portable systems for the nucleic acid-based detection of bacterial pathogens.

## 2. Nucleic Acid-Based Detection

Despite vast improvements in modern-day pathogen detection techniques, the tried and true culturing and plating method still remains the standard method of detection. This technique involves culturing and measuring the growth of individual viable microorganisms using either non-selective media, such as trypticase soy agar, or selective media specially formulated to detect a particular bacterial species. At lower detection levels, however, this method would require a lengthy pre-enrichment step to increase the numbers of viable target bacteria before detection could be conducted. Detection is mainly through enumeration by ocular inspection, which leads to sources of uncertainty due to human variations in sampling and measurements [20]. Due to the low throughput, time consuming and labor intensive process of colony enumeration, this method exhibits low potential for integration and miniaturization into micro total analysis systems. Though several bacterial colony counters have been proposed to automate and standardize this process [21-23], culturing remains a time-consuming process and the required high-quality imaging equipment and software are expensive and cumbersome for integration into a portable system. The recent push for reliable, rapid detection techniques is prompting researchers to explore alternative methods, particularly for detection of bacteria with slower generation times such as the gastroenteritis-causing *Campylobacter* species, which require a minimum of 3 – 4 days for full-confirmation [24].

Nucleic acid-based methods in pathogen detection are promising in their rapid results, high specificity, and low detection limits of up to, in theory, a single cell. Developed in the mid-1980s, nucleic acid-based technology quickly achieved widespread use in the field of pathogen detection, with a particular focus in polymerase chain reaction (PCR) assays that were developed to detect virtually every clinically relevant bacterial pathogen [1]. In the past decades, our understanding of DNA has grown considerably, with currently 788 fully sequenced microbial genomes [25]. The versatility of nucleic acid-based methods allowed for the design of specific probe sequences, typically on the order of 10 to 30 base pairs in length, to target antibiotic resistance genes as well as for sub-typing of bacteria. DNA is an excellent vehicle for signal transduction due to its characteristic negative charge, and in addition to the typical optical and mechanical measurements, pathogen sensors are often designed to quantify hybridization events between analytes and probe DNA based upon electrical measurements as well. Since these methods target nucleic acids, however, they do not indicate the viability of the target pathogen, so care must be exercised when performing these tests. On the other hand, there are situations where the detection of hibernating or non-viable pathogens is favorable, particularly when aiming to detect unculturable cells [26-28] or to quantify antibiotic effectiveness [29-31]. Nucleic acid-based techniques have a higher sensitivity, therefore requiring a higher level of quality control to prevent contamination, elevating the importance of effective sample preparation to a critical step for successful detection. Consideration of contamination, inhibitors in the specimen sample, and DNA degradation due to unfavorable conditions must be accounted for in the sensor design to help reduce the incidence of false positive or false negative results.

**Figure 1.**
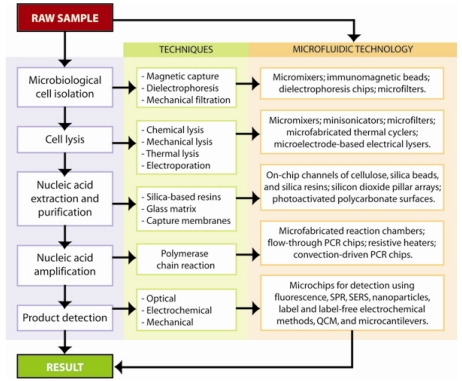
Diagrammatic representation of the processing flows in nucleic acid-based chip detection, the traditional techniques and translation into microfluidic technology.

Modern advances in micro- and nanofabrication technology have led to the development of a wide range of nucleic-acid based biosensors that capitalize on the new capabilities of microfluidic technologies and micro total analysis systems in order to reduce reagent and power consumption, enhance analytical performance, and enable portability. These lab-on-a-chip devices incorporate multiple laboratory processes in a semi-automated and miniaturized format. Many of these technologies have been extensively studied [32], successfully commercialized, and are currently widely used in clinical and research laboratories. Nevertheless, portable biosensors systems for point-of-care diagnostics and on-site field testing are still in the infancy stage. Current portable systems tend to be costly and require additional resources as well as skilled operators, therefore rendering the technology unsuitable for point-of-care testing, especially in resource-poor regions such as Africa, Asia, and Latin America that would benefit the most from the development of these platforms [33,34]. Performance of a biosensor platform in the third-world is challenged by the absence or scarcity of trained workers, electricity, equipped laboratories, transportation, and refrigerated storage [34]. Specific areas that need to be addressed during further development include sample pre-treatment, long term storage of reagents, ease of use, and costs [35]. Point-of-care biosensor systems, particularly those utilizing disposable cartridges, must direct some attention towards the development of environmentally-friendly chemicals and materials [34]. Though multiple sensors and assays have been developed for lab-on-a-chip nucleic acid-based detection, few systems have successfully integrated all the necessary sample preparation, sample handling, and detection components into a single automated, portable platform with raw-sample-to-result capabilities. An overview of the translation of traditional microbiological techniques into microfluidic technology is represented in Figure 1.

## 3. Microfluidic Nucleic-Acid Based Pathogen Detection Systems

Proposed by Manz *et al.* [36] in the early 1990s, micro total analysis systems (μTAS) are integrated miniaturized platforms composed of multi-step sample preparation and detection systems on a single chip that has raw-sample-to-result capabilities – the quintessential “lab-on-a-chip” concept. μTAS systems have experienced rapid growth and development since the completion of the human genome project. The driving force for miniaturization has always been improvement in performance. At the microscale, faster, higher-throughput analysis using parallel systems can be achieved due to a combination of larger surface-to-volume ratios, reduced separation times, shorter diffusion paths, and more efficient reactions. This points not only to the potential for low costs associated with reduced reagent consumption, but also to the ability to analyze smaller samples that were previously insufficient in size. In terms of DNA detection, polymerase chain reaction (PCR) and various other sensing schemes have been successfully carried using integrated microfluidic systems [32]. DNA sequencing and genotyping have been achieved through advances in microchannels technology and capillary array electrophoresis. Complete nucleic acid-based analysis involves complex processes, such as cell concentrating and capture, cell lysis, nucleic acid purification, amplification, and final detection.

### 3.1. Materials and fabrication for microfluidi*c* sensors

The first generation of microfluidic devices built in the early 1990s were mainly fabricated on silicon and glass substrates by borrowing technology generated by the massive growth of the semiconductor industry, such as photolithography and etching techniques. Investigations into new unconventional substrate materials for biocompatible microfluidics, led to interest in ceramics, hydrogels, and even paper. To meet the demand for cheaper, more versatile alternatives, however, researchers began to explore the use of polymeric materials in microfluidic technology. Biocompatible polymeric materials can be easily selected for DNA-related analysis, since the magnitude of electroosmotic flow is significantly lower than that of glass and fused silica in similar pH environments, lessening the need for further coating treatments of the microchannel surfaces to prevent nonspecific adhesion [15]. However, different surface chemistries may need to be investigated in order to reduce unwanted polymer absorption of reagents and optimize the analysis system. One other drawback is the incompatibility of most polymers with a range of non-aqueous solvents commonly used in chemical production and drug discovery [19], although for most nucleic acid-based detection purposes this issue does not pose a problem.

**Figure 2.**
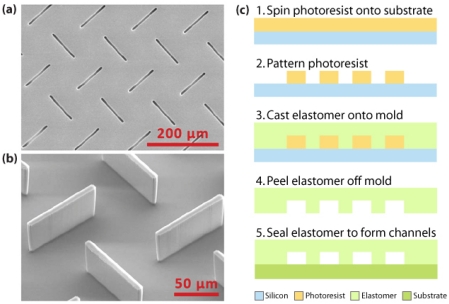
SEM micrograph of (a) PDMS mold for plastic casting and (b) the epoxy chip fabricated by casting (reprinted with permission [37] ^©^ 2007 Springer); (c) Schematic of the casting method showing an elastomer material poured over a molding template, peeled off, and sealed with an appropriate substrate, such as glass or silicon, to form microfluidic channels.

There are two main methods to micromachine polymers. The cheaper, more commonly used method is replication, which involves methods such as hot embossing, injection molding, and casting to transfer a pattern from a precision template or master to a polymer substrate. This master mold can be made from a variety of different materials: glass, silicon, metals, and more recently, high-aspect-ratio photoresists. Hot embossing is a simple procedure that involves heating the polymer to slightly above its glass transition temperature and applying it to a master under vacuum to form a polymer device with high structural integrity [38]. Though this process is quick and relatively inexpensive, it cannot be a fully automated process [39]. Injection molding is one of the most well-known technologies where heated polymer pellets are injected at high pressure into a mold to replicate features at rapid rates and high-volume production [40]. Casting is by far the most widely used technique in the academic world. It is an easy, low-cost process of pouring a polymer material over a molding template and curing, after which the soft elastomer copy can simply be peeled off the mold and used [41], as seen in Figure 2. Most commercial devices fabricated today are made from polymers such as polycarbonate (PC) and polymethylmethacrylate (PMMA), while polydimethylsiloxane (PDMS) is still widely used in research [19]. The more versatile, direct fabrication methods, such as laser ablation, optical lithography, and X-ray lithography, tend to be more suitable in a research setting due to customizability of each individual device. Laser ablation, a precise approach that can achieve submicron features, uses the energy of a pulsed laser to disrupt polymer bonds and remove polymer fragments from the ablated region to form a clean cut surface; however, the laser light may induce unwanted surface modifications on the polymer material [7,42].Optical lithography techniques of fabricating microfluidic channels include the patterning or layering of polymer and sacrificial material, where the sacrificial material is subsequently removed using appropriate solvents. Stereolithography is another optical technique where focused laser light is used to photocure a liquid polymer, but this process tends to be slow and tedious [7]. In order to push the envelope on minimum feature sizes that can be realized in polymer devices, the use of X-ray lithography for patterning has also been investigated [43].

All microfluidic devices require a tight bond or seal of the channel or chamber to form an enclosed structure, and a variety of material-dependent techniques have been used to achieve reliable containment of the sample fluid. For PDMS-based microfluidic fabrication, O_2_ plasma is commonly used to activate the PDMS surface to produce polar groups (Si-OH) and when two activated surfaces are brought in close proximity, an irreversible bond is formed capable of withstanding high pressures. Other bonding methods include lamination, thermal bonding, ultrasonic welding, and the use of adhesives.

### 3.2. On-chip fluid and reagent handling

Precise fluid control and flow stability in a microfluidic-based system is critical for successful DNA detection. As a sensitive detection system, the introduction and maneuvering of any fluids must be done with extreme care so as to prevent bubble formation within the channels or chambers. Though bubbles can be used as an actuation mechanism for various applications [44], the presence of undesired bubbles can adversely affect or block sample flow, causing detection failures, particularly in highly-sensitive optical detection schemes. Some research has been conducted in the implementation of bubble traps as a prevention scheme in microfluidic systems. There has been extensive research in microfluidic fluid-handling for the manipulation of on-chip fluids via pumps, valves, and mixers, discussed in the following.

#### 3.2.1. Microfluidic Pumping

One of the earliest micropumps were developed by Smits in the 1980s for the controlled delivery of insulin to maintain the blood sugar levels of diabetics [45]. Since then, a variety of different pumping mechanisms have been explored for chemical and biological analysis applications, with attempts to make improvements in the areas of pressure generation, cost, power consumption, biocompatibility, and reliability. Some microfluidic applications where pumping serves a vitally important role include cellular capture and separations [46,47], DNA purification [48], and flow-through PCR [49]. Microfluidic pumps currently employ a variety of different actuation mechanisms: thermopneumatics [50,51], electrostatics [52-54], piezoelectrics [55-57], electromagnetics [58-60], and hydrogels [61,62], among others. Some microfluidic pumps focus on controlled direction and delivery of micro- and nanoliter solutions over long periods of time, while others seek to achieve high pumping volumes at low power. Thermal and electrolytically-generated bubbles have been investigated for their utility in miniaturized pumps, microfluidic dosing experiments [63], and are favorable due to simple fabrication and ease of control. The disadvantage of thermal production of water vapor bubbles is the risk of denaturating biological molecules due to overheating [64]. In addition, the electrolytic production of bubbles has been shown to be far more energy efficient than thermal bubble generation [65]. Gravity, vacuums, wicking and capillary action have also been widely used to motivate fluids [17] and are generally favored in portable systems due to their low power consumption. Despite extensive research, however, there are still limited μTAS systems with on-board micropumps in existence, since most systems still rely on manual pipetting, syringe pumps, or induced electroosmotic flow for liquid transport.

#### 3.2.2. Microfluidic Valving

In order to meet the complex plumbing requirements set forth by μTAS systems, particularly for high-throughput multiplexed systems where a multitude of different samples and reagents need to be activated and inactivated with precise temporal control, microvalves have been investigated for a variety of applications. Typical valves at the macro-scale use hydraulic, pneumatic, manual, or solenoid activation [17], most of which can be easily rescaled to our microscale needs. Many of the actuation mechanisms and methods employed for microvalve construction draw upon the same principles used by microfluidic pumps [66-69], and therefore have similar associated advantages and disadvantages. Some actuation mechanisms heavily rely on the properties of the working fluid (i.e. electrokinetic manipulations), and can therefore be too specific in its application. For higher versatility, microfabricated mechanical valves are a robust alternative. Other valving mechanisms include temperature-control of paraffin [70], manipulation of the hydrophobicity properties of chemically modified elastomer for low pressure valving [71], electrochemical generation of microbubbles [72], and the usage of thermally-responsive polymer solutions [73]. The low power consumption need of a portable system tends to favor pneumatic or externally-coupled mechanical mechanisms. Some of the performance criteria to keep in mind when designing valves are size, dead volume, channel dimensions, actuation pressure, power consumption and scalability [67].

#### 3.2.3. Microfluidic Mixing

The challenge of mixing of reagents and samples in a small volume can be difficult to overcome due to low Reynolds numbers, and diffusion and convection limitations at the microscale. A wide range of methods have been proposed to achieve efficient mixing of two or more fluid streams. At the macroscale, magnetic stirrers are the conventional solutions for homogenous mixing, and this idea has been adapted to the microscale using a miniaturized magnetic stir bar [74,75]. Active mixing methods, such as those based on electro-hydrodynamic, magneto-hydrodynamic, pressure perturbations, ultrasonic, centrifugal, and electrophoretic principles, often require moving parts and external power sources, which may reduce the feasibility of a portable system [17]. Mixing from oscillatory motion has been investigated with the use of piezoelectrics [56], gas bubbles [44,76] and magnetic microspheres [77]. Passive mixers are typically designed to reduce diffusion lengths through increased surface area and the creative manipulation of fluids by the positioning of special microfabricated structures. Some notable passive mixers in literature include the kneading of fluids through the positioning of herringbone grooves on the channel surfaces [78], continuous-flow mixing capable of reaching 95% mixing completion in 15 milli-seconds, and innovative devices modified with Tesla structures [79] and J-shaped baffles [80]. Although passive mixers enjoy the advantages of low power consumption and the lack of wear and tear associated with mechanical parts, the complex channel topology needed to achieve efficient passive mixing can often be difficult to microfabricate. Appropriate micromixing technology should be chosen based on degree of mixing required, fluid volumes, power consumption, ease of fabrication, and feasibility.

## 4. Filtration and Separation of Bacterial Cells

The current selection of nucleic acid-based biosensors with target detection of a highly specific DNA signature dictates the need for simple and effective methods of obtaining high-quality DNA. For the majority of biosensing applications, the starting samples consist of tissue, blood, environmental, or food samples [81] and need to undergo careful sample preparation for sensitive detection due to trace or low-abundance species. Although many of the assays based upon polymerase chain reaction (PCR) are fairly robust, a variety of contaminants can inhibit amplification and diminish the success of such analytical instruments. In order to circumvent this problem, target cells must first be extracted and purified from a raw sample through a variety of cell separation and capture techniques. Cell concentrators increase the concentration of microorganisms through gentle means, so as to preserve specific activity or viability, and are important to help increase the sensitivity and strength of the final detection signal [82]. Also, raw samples taken from blood, soil, water, or food are often large in volume for microfluidic analysis, and this discrepancy in volumes makes concentration necessary due to time constraints and the need for rapid detection. The volume analyzed in a typical microscale pathogen detection device ranges from a couple picoliters to, at most, a few microliters. Cell separation is important for separating target cells from contaminants in the raw sample. The three main techniques for cell manipulation involve the use of magnetic, electrokinetic, and mechanical principles.

**Figure 3.**
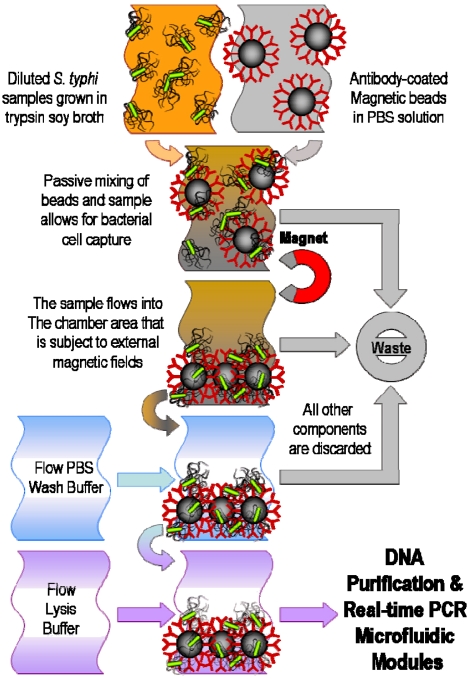
The process flow for microfluidic immunomagnetic cell isolation, buffer wash, and subsequent chemical lysis of *Salmonella typhi* using antibody-coated paramagnetic beads and external magnetic field capture.

Magnetic manipulation techniques typically use magnetic particles that can selectively attach to cells of interest through the use of antibodies and other linking chemistries, and use magnetic field gradients to capture the bead-cell conjugates, as illustrated in Figure 3. Investigations into continuous flow separations [83,84] and matrix-based manipulations [85] using magnetic capture have been promising. *E. coli* has been shown to be magnetically separated from PBS and whole blood on an integrated microfluidic device consisting of a chaotic mixer, incubation channel, and a capture channel [86]. The magnetic method is clean, versatile, and non-invasive, and with advances in magnetic bead materials and chemical modification techniques, the technique has the potential to become increasingly efficient and easily integrated into a portable system [18].

Cell manipulation using dielectrophoresis (DEP) takes advantage of the intrinsic dielectric properties of cells and their response to electric fields, and has been extensively studied on microscale devices [87]. A DEP chip fabricated from acrylic has been reported by Huang and colleageues to separate *B. cereus*, *E. coli*, and *L. monocytogenes* from blood [88]. Using DEP microchip technology, live cells have been shown to be separated from dead cells through differences in cellular dielectric properties at differing states of viability [89]. In addition, single-cell trapping has been achieved using DEP in conjunction with laser-trapping forces [90]. Mechanical cell separations have been achieved using microfilters [91], microwells [92,93], and surface-modified microchannels [94,95]. Microbial cells have been concentrated using size-dependent filter-based microfluidic devices [91], which are typically rapid and highly efficient, though lacking in selectivity. For portable point-of-care devices, low cost and power consumption is necessary, without sacrificing on sensitivity, and magnetic bead-based separations have shown considerable promise in this area.

## 5. Pathogenic DNA Extraction and Purification

Upon cell capture and isolation from the raw sample, cell lysis is necessary to release the nucleic acids for further analysis. Among the various lysis methods, chemical lysis is most common. Chemical lysis can be easily incorporated into an integrated microfluidic design with methods such as on-chip mixing of captured cells with sodium dodecyl sulfate or guanidinium thiocyanate [96] and hydroxide electro-generation-induced cell poration and lysis [97]. Unlike mammalian cells, the efficient lysis of certain bacteria for DNA extraction can be more challenging. Gram-negative bacteria is commonly treated with alkaline buffers or guanadinium thiocyanate, whereas gram-positive bacteria is more difficult to lyse often requiring multistep methods, though heating in the presence of chelating resins, sometimes with beads, has been shown to be effective [98]. Heat-based techniques, such as freeze-thaw or freeze-boil methods [99] are also available, and pulsed laser irradiation of carboxyl-terminated magnetic beads [100] has been reported for on-chip pathogenic DNA extraction. However, most thermal methods are seldom employed due to likelihood of denaturation due to high heat. Electrical pulsing methods have also been incorporated into microfluidic chips to electroporate cells [101]. Mechanical disruption methods, such as sonication, release cellular components into solution but often require more energy but have been demonstrated in microscale devices [102,103]. High-frequency sonication uses piezoelectric materials to generate pressure waves that disrupt cell membranes, and though effective against hard-to-lyse cells, this method generates considerable amounts of unwanted heat and free radicals [98].

**Figure 4.**
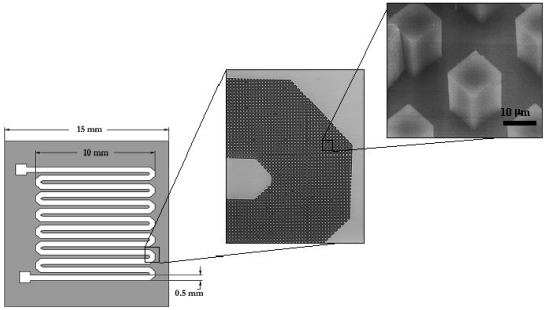
Schematic representation and SEM micrographs of channels containing microfabricated silica pillars used for microchip DNA purification (reprinted with permission [48], ^©^ 2003 Elsevier Science B.V.).

The traditional method of purifying DNA is performed via proteinase K digestion in the presence of detergents, phenol-chloroform extraction, and concentration by alcohol precipitation [98]. One of the most common modern techniques for DNA purification is through chemical lysis followed by capture using silica-based resins. DNA in chaotropic salt-containing buffers such as those containing guanidinium or sodium iodide salts, preferentially bind to silica surfaces, whereas other macromolecules such as proteins and lipids remain free in solution [104,105]. These unwanted components are traditionally removed using centrifugation and alcohol washing steps using commercially-available kits. However, the fact that they are usually based upon particulate matrices presents challenges to integration onto μTAS devices. While incorporation of silica-based resins into a microfluidic device has been reported [105,106], new innovative silica pillar arrays (see Figure 4) have also been investigated for microscale DNA purification [48] which circumvent the problems associated with filling channels with binding matrices after microfabrication.

## 6. Pathogenic DNA Detection

### 6.1. Polymerase chain reaction amplification and detection

**Figure 5.**
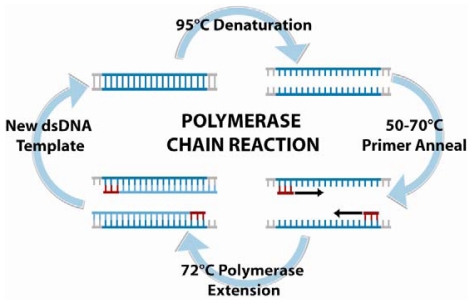
Polymerase chain reaction (PCR) is an amplification-based technique for DNA detection. The standard protocol involves raising the temperature of the reaction to 95 °C to separate the DNA strands, lowering to the annealing temperature for the oligonucleotide primers to hybridize, and then raising to the optimal DNA polymerase temperature 72 °C for primer extension. This process is repeated cyclically, creating many copies of the target sequence.

For smaller test samples, DNA amplification is often necessary to obtain a sufficiently strong DNA-detection signal. PCR is a three-step amplification process, depicted in Figure 5, first introduced in 1985 by Saiki *et al.* [107].

The principle of PCR is based on the isolation, amplification, and quantification of a short but unique DNA sequence present in the target bacteria's genetic material. For conventional PCR, forward and reverse primers are used to amplify the target sequence, and subsequent gel electrophoresis in conjunction with DNA-binding fluorescent dyes allows visualization of the result. Though this technique is significantly less time-consuming than the culturing and colony counting method, the typical laboratory PCR procedure time frame still ranges from 5 to 24 h, without taking enrichment times into account. Since bacterial nucleic-acid-based detection is mainly DNA-based, reverse-transcriptase PCR (RT-PCR) are less frequently used. On the other hand, real-time PCR typically employs an automated system and special fluorescent probes that track the amplification during the thermal cycling. Common probes used in these assays include the dual-labeled TaqMan^®^ probes, hybridization Light-Cycler probes, intercalating SYBR Green^®^ dye, Molecular Beacons, or Scorpions™. This technique offers a variety of advantages, including increased sensitivity, speed, broader dynamic range, and higher throughput. The major limitation of PCR points to the high cost of instrumentation and reagents, but the technology is highly amenable to miniaturization for applications in portable biosensing and point-of-care diagnostics.

Since the introduction of the first PCR chip in 1995 by Northrup and colleagues [108], a multitude of PCR microfluidic technologies have facilitated a variety of improvements in microfluidic DNA amplification, such as smaller volumes, faster ramping rates, lower manufacturing costs, and higher integration. Successful chip-based DNA purification and PCR requires manufacturing of the detection microchips, as well as development of a platform to perform the necessary thermal cycling and DNA detection measurements. In order to amplify DNA with high specificity and high throughput, the cycling temperatures in PCR microfluidics must be precisely controlled to achieve desirable temperature kinetics for the denaturation, annealing, and extension steps. For single-chamber PCR thermal cycling, investigators have employed multiple techniques, including infrared light [109], thermoelectric heater-coolers [110], and resistive electrodes [111]. In addition to changing the temperature of the entire reaction chamber, other researchers have developed flow-through PCR devices, in which the sample is passed through different thermal regions on a serpentine or circular chip [49,111,112]. Convection-driven PCR microfluidics uses buoyancy forces to drive the sample fluid forward between the temperature zones [19], and has also been shown to be capable of rapid DNA amplification. For high-throughput analysis, multi-chamber PCR microfluidic devices have been constructed for parallel processing [113-115], though careful design is necessary to ensure temperature uniformity, reliability, and repeatability across the different chambers [19]. Methods of subsequent DNA detection are primarily grouped into optical, electrochemical, and mechanical techniques, as discussed in the following.

### 6.2. Optical methods in nucleic acid-based detection

One of the primary methods of observing and quantifying DNA is through the use of optics. Traditional laboratory methods of quantification have utilized the specific absorption of ultraviolet (UV) light at 260 nm by DNA. For most biosensing applications, techniques have mostly focused on the use of fluorescent dyes and, more recently, quantum dots. However, the past decade has shown an escalating surge of interest in techniques such as surface plasmon resonance (SPR), surface-enhanced Raman scattering (SERS) spectroscopy, interferometry and colorimetry.

#### 6.2.1. Fluorescence-based detection

Fluorescence is the optical technique most commonly employed due to its high level of sensitivity and low background noise. Fluorescent dyes can bind to DNA non-specifically through general interactions, or attach directly to specific locations on a DNA molecule, and the resulting signal can be easily detected with an appropriate imaging apparatus. The first label used in 1953 was fluorescein for the immunofluorescence of DNA, with rhodamine following not long after, both dyes utilizing isothiocyanate reactive groups to conjugate to the free amine groups on nucleic acids [116]. Ethydium bromide, one of the original non-specific DNA dyes, was first described for DNA quantification in 1967 [117], and is still commonly used for DNA visualization during gel electrophoresis. Since then, several sequence-independent dyes exhibiting high fluorescent signals when bound to DNA have been developed, including the YOYO and TOTO dyes [118], PicoGreen, and SYBR Green [119], among many others. The limitation of conventional fluorescent dyes lies in the background fluorescence, as well as the photobleaching and time degradation of fluorophores.

**Figure 6.**
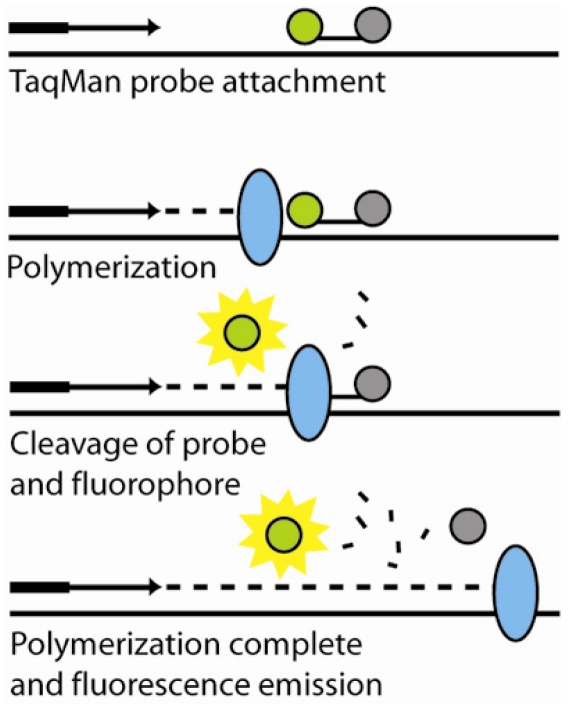
The TaqMan assay, also known as the 5′ nuclease assay utilizes a third oligonucleotide labeled with a fluorophore (green) and quencher (gray), in which the fluorophore is quenched due to FRET conditions. During the reaction, the DNA polymerase (blue) degrades the probe, separating the fluorophore and quencher, allowing for fluorescence emission to occur.

An interesting advancement in optical DNA detection was the development of fluorescent resonance energy transfer (FRET), which utilizes a distance-dependent phenomenon that occurs when a donor fluorophore and an acceptor chromophore (quencher) are in close proximity (typically 5 – 10 nm) and excitation energy is transferred from the flourophore to the quencher, thus preventing fluorescence emission. Multiple detection techniques have been devised to harness this effect, including molecular beacons and 5′ nuclease (Taqman^®^) real-time PCR detection. Molecular beacons are designed to preferentially base-pair with itself, forming a stem-loop structure that brings the flourophore and quencher into close proximity. A probe sequence in the loop region on the molecular beacon can hybridize with the target DNA, causing the step-loop structure to open, separating the fluorophore and quencher and resulting in fluorescence. Molecular beacons have been patterned and immobilized on solid supports due to their potential for label-free, real-time detection in the DNA array format. In the case of the Taqman^®^ real-time PCR technique, the modified oligo-probe is degraded during the primer extension of PCR amplification, releasing the fluorophore and quencher into solution separately, as shown in Figure 6.

Both methods are dependent on proper probe design to achieve sequence specificity to obtain a quantifiable fluorescent signal. Other types of probes used in DNA fluorescent detection include scorpions and light-up probes. Similar to molecular beacons, scorpions are linked to the primer but cannot be fully copied during PCR due to the presence of a blocking molecule, which allows it to be faster and more efficient than molecular beacons, while remaining sensitive enough to detect single-base mutations [120]. On the other hand, light-up probes are peptide nucleic acids tethered to a dye molecule that binds to the target DNA upon probe hybridization, and essentially “lights up” the fluorescence signal. These probes do not rely on the FRET process, and are capable of hybridizing more quickly and strongly than oligonucleotide probes [8].

**Figure 7.**
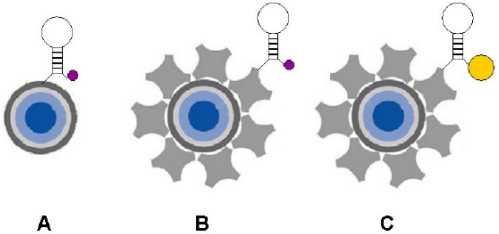
A representation of the three different quantum dot molecular beacon labeling strategies is shown. Carboxyl-modified quantum dots (blue core) were modified with amino-labeled molecular beacons (A) while streptavidin modified quantum dots (blue core dot with surrounding gray streptavidin molecules) were modified with biotin-labeled molecular beacons. Both dabcyl and Iowa Black FQ quenchers (small purple circles) were used, as well as 1.4nm Nanogold (gold colored circle), (reprinted with permission [122], ^©^2006 Elsevier Ltd.)

In order to enhance the signal-to-background ratio of the fluorescent signal, a variety of different alternative techniques, such as quantum dots and fluorescence resonance energy transfer (FRET) have been explored. Quantum dots are advantageous in that they fluoresce throughout the visible and near infrared and can be excited with a single blue UV excitation source. In addition, they are resistant to photobleaching and have brighter, narrower emission bands so that theoretically, as many as 20 quantum dot reagents could be individually detected using narrow band-pass filters [116]. There have even been investigations in using quantum dots in conjunction with molecular beacons (see Figure 7).

Multiple binding chemistries are readily available to for attachment of DNA molecules to quantum dots. Conversely, quantum dots are typically larger than conventional dyes, and have been suggested to negatively affect probe-target interactions and in some cases cause steric hindrance [121]. There exists a vast selection of chemistries and probes available for the fluorescent detection of non-specific and specific nucleic-acids, many of which are highly adaptable to miniaturization schemes for lab-on-a-chip applications. Modern-day fluorophores display excellent fluorescence, so there is little pressure for incremental improvement in this arena. However, with the continued push towards smaller instrumentation and sample sizes, chemical and photostability for robust sample preparation, shipping, storage, and manipulation becomes vitally important [116]. And though quantum dots are highly fluorescent and photostable, the problematic issue with size compatibility is still unresolved. The movement towards obtaining data from single-molecule fluorescent detection measurements signifies a need for a highly-fluorescent, photostable fluorophore capable of low-level multiplexed detection.

In the field of portable fluorescence detection, the design and integration of miniaturized excitation and emission sources for microchip devices has been challenging. Bubble formation becomes a major concern during PCR because they scatter light and can significantly reduce the sensitivity of an instrument relying on optical detection. Traditional excitation is done using bulky, bench-top sources, such as lasers and mercury lamps [105,106,123] and detection is typically accomplished with microscope-based CCD cameras, laser scanning microscopes, or other large instrumentation that severely inhibits portability [105,106,123] due to size and power consumption. In contrast to these larger systems, light-emitting diodes have been applied as low-power excitation sources, in conjunction with smaller footprint detectors such as photodiodes and miniaturized photomultiplier tubes [32,104,124,125]. Miniaturized spectrometers have also been proposed, enabling detection of a continuous fluorescence spectrum, thus allowing multiplexed detection with the use of different labeling dyes. End-point detection conventionally involves an after-PCR fluorescence measurement, followed by processing steps of gel or capillary electrophoresis [126]. Real-time detection of PCR products has an advantage over end-point detection due to its potential for faster detection due to the ability to observe the yields in real-time rather than waiting for the entire PCR cycling process to complete, and also requires less complex machinery. From real-time PCR results, the initial DNA concentration can be extracted, offering more reliable results and could provide an important piece of data in analyzing the degree of pathogen contamination in raw samples.

#### 6.2.2. Surface plasmon resonance

**Figure 8.**
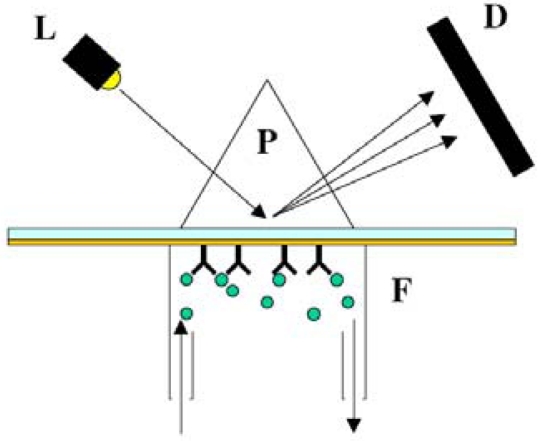
Surface plasmon resonance (SPR) is typically performed using an optical light source (L) coupled to a thin metal surface (S) through a prism (P). During experimentation, changes in the refractive index are measured by a detector (D). A flow cell (F) is commonly used to bring fluids into contact with the thin film, allowing for binding to other molecules on the film surface, (reprinted with permission [133]).

Surface plasmon resonance (SPR) is an optical technique the reports changes in the refractive index of a metal film that occurs during adsorption of target DNA molecules to that film (14). For most SPR-based biosensors, the metal film is composed of gold, and DNA probes are assembled on this gold surface such that the target binding event results in a change in measured refractive index [127-130]. The SPR system, illustrated in Figure 8, is particularly useful in determining binding and dissociation kinetics, and has even been shown to be sensitive enough to detect DNA mismatches [8].

By interfacing with imaging technology, SPR spectroscopy allows for studies of DNA assembly, hybridization, and protein-DNA interactions on bio-functionalized chips. Since SPR alone is often not sensitive enough to accurately monitor binding events of low molecular weight molecules and low packing density molecules, fluorescent tagging is often performed in conjunction with SPR in a technique called surface plasmon field-enhanced fluorescence spectroscopy (SPFS) [14]. Recent developments of multiplex SPR systems have been explored [131,132], and though the use of SPR for DNA detection is not as widely reported as alternative methods, the high sensitivity of this technique makes it a viable and useful option of nucleic acid-based sensing on microchips.

#### 6.2.3. Raman detection

Raman spectroscopy allows for measurement of a “chemical fingerprint” for analyte identification by studying the vibrational, rotational and other low-frequency modes in a system. Typically, laser-based monochromatic light excitation is used to excite in the visible wavelength range [16]. Surface-enhanced raman scattering (SERS) techniques have been applied by a number of investigators for sequence specific DNA detection [134,135]. Multiplexed SERS detection was demonstrated by Docherty and colleagues using three dye-labeled oligonucleotides on microchips [136], though complex computational analysis is still needed for improved peak shapes. A major advantage of Raman methods lies in the fact that water is virtually Raman transparent, and therefore adsorption by water molecules does not pose a problem during detection. The technology is easily miniaturized, and a variety of surface and resonance enhancement techniques can be utilized to improve sensitivity [16].

### 6.3. Electrochemical methods in nucleic acid-based detection

**Figure 9.**
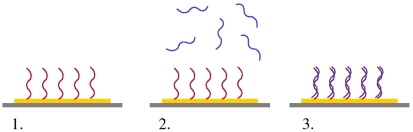
A general strategy for electrochemical DNA detection begins with immobilization of a ssDNA capture probe on an electrode surface: (1) After probe immobilization, baseline electrical measurements are taken and then target DNA is added; (2) Target DNA is allowed to hybridize with the capture DNA; (3) Another set of electrical measurements are made to detect the electrode changes caused by DNA hybridization. Detection can often be further enhanced by modifying the DNA with electroactive compounds or metallic nanoparticles, i.e., indirect detection [133].

Many of the electrochemical methods for DNA detection are comparable to fluorescence techniques in their simplicity, high sensitivity, low cost, and compatibility with microfabrication technology. An added advantage of electrochemical methods is their potential for portability, whereas fluorescence methods typically employ bulkier instrumentation. A variety of different electrochemical techniques are used to detect DNA hybridization, some with labels such as electroactive hybridization indicators, enzymes, or nanoparticles. A general strategy for electrochemical DNA detection is shown in Figure 9. The wealth of immobilization chemistries available for patterning probe sequences on a variety of electrode substrates allows for detection to be accomplished using inexpensive electrochemical analyzers. On the other hand, a variety of different label-free electrochemical nucleic acid sensors have also been reported [137], such as capacitance-based sensing of changes in the biolayer dielectric [138]. The three main detection signals that are measured during electrochemical detection are: current, potential, or impedance.

#### 6.3.1. Labeling techniques

Electroactive hybridization indicators, such as cationic metal complexes or organic compounds that recognize the DNA helix structure intercalate selectively into double-stranded DNA, are extremely common [8, 139] for electrochemical detection. Despite its popularity, this technique does not have the specificity to detect mutations in DNA sequences [8]. Enzymatic labels are attached to target or probe nucleic acids directly for highly specific detection: when enzyme-labeled DNA reacts or hybridizes with immobilized probes or single-stranded DNA, the rise of amperometric current has been shown to be proportional to the number of hybridization strands. Labeling with soybean peroxidase (SBP), a thermostable enzyme, has been demonstrated to provide measurements in real time without any need for a washing step. The three-component sandwich assay is a variation on the enzyme approach where a label is designed to a probe-target complex that eliminates the need to directly modify the target strand with the label, and paves the way for reliable multiple-target detection. Colloidal gold nanoparticles have also been incorporated into the sandwich assay format for significant signal enhancement [140,141], pushing the detection limit of PCR amplicons to as low as 0.8 femtomoles of DNA. Willner and colleagues have added a second dimension to the nanoparticle approach by developing quantum dot CdS particle-labeling of DNA to provide photoelectrochemical detection of hybridization events. Operation of the sensing scheme involves exposure of the aggregate to visible blue light to trigger a current between the CdS nanoparticle aggregate and the gold electrode [35]. Other electrochemical sensing approaches have involved materials such as magnetite [142] and carbon nanotubes [143].

#### 6.3.2. Amperometric detection

One of the most common electrochemical detection methods, amperometric detection senses the oxidation or reduction of an electrochemically active analyte at the electrode interface, which is typically constructed out of platinum, gold, and carbon. The operation of this sensor relies on the linear relationship between analyte concentration and measured current. In cases where direct electron exchange cannot occur between the electrode and the biomolecules, special mediators called redox mediators are required to reversibly exchange electrons between the sensor and enzyme [144]. One demonstration of an amperometric-based flow-through immunofiltration assay has detected between 100 – 600 cells per mL of *E. coli* within 30 minutes [145]. Though the amperometric method is capable of detecting cells directly using antigen-antibody biorecognition elements, investigations of nucleic acid-based amperometric detection of microbial contamination in food and water have also been reported [146].

#### 6.3.3. Potentiometric detection

Potentiometric methods yield a logarithmic concentration response, enabling the detection of extremely small concentration changes with continuous measurement capabilities, but are the least implemented in biosensors, possibly due to lower selectivity and higher limits of detection in certain environmental samples. Modified ion-selective field effect transistors (ISFETs), devices consisting of a p-type silicon substrate with two n-doped regions separated by a short distance (gate) and covered by an insulator layer, have been shown to use the semiconductor field effect to detect biological recognition events [147]. However, incompatibility of materials with immobilization techniques, complicated fabrication and packaging, along with device instability impose severe limitations on this technology [11].

#### 6.3.4. Conductimetric and Impedimetric Detection

Electrochemical impedance spectroscopy (EIS) is a powerful technique that applies a small amplitude sinusoidal excitation signal to a given system and measures the response in either current, voltage, capacitance, resistance, or some other signal form [11]. First applied to the detection of bacteria biomass in foods over ten years ago by measuring electrical impedance changes due to bacterial growth, the method is now widely accepted and applied [1]. More recently, disposable conductimetric biosensors with a detection limit of 83 CFU per mL have been reported that use polyclonal antibodies against *E. coli* [148]. The advantage of EIS lies in its label-free detection, however, it has a limiting factor of poor sensitivity as compared to other traditional methods [1] and careful circuit design must be done to ensure reliability [149]. To combat this low sensitivity, high density microelectrode arrays [150], sandwich assays [151], and nanowires [152] have been implemented for pathogen detection.

### 6.4. Mechanical methods in nucleic acid-based detection

Fluorescence, amplification and electrochemical-based techniques all exploit various intrinsic properties of DNA to create a measurable signal. One of the most basic properties of DNA is mass. Like any molecule, DNA possesses a certain mass that can be measured directly using frequency-based detection methods. The laws of physics dictates that solid rigid objects have inherent resonant frequencies that can be shifted by attaching an additional mass and from this frequency shift, one can mathematically extract the associated change in mass.

#### 6.4.1. Quartz crystal microbalance

The quartz crystal microbalance (QCM) is an instrument that utilizes a piezoelectric quartz crystal that can be vibrated at high frequencies with an electrical current to perform frequency-based measurement of DNA mass. The QCM can easily be converted to a DNA sensor by immobilizing probe DNA on the surface of the quartz crystal, and subsequent hybridization to target DNA will cause a change in resonant frequency. DNA hybridization events have been detected using QCM, with enough selectivity to discriminate between complimentary and non-complimentary target DNA, proving the capability of distinguishing between variant DNA sequences [153,154]. QCM-based system have also been used as end-point measurements for PCR-based detection systems via immobilization of the capture probe on the quartz crystal [155]. In some instances, target DNA have been modified with secondary compounds, such as gold nanoparticles, for the increased mass and associated increased measurement sensitivity. Using 50 nm diameter gold particles, the sensitivity of a system couldbe increased to between 10^-15^ and 10^-16^ M of DNA [156]. One major limitation with QCM is the difficulty of incorporating multiplexed detection of multiple samples, although there have been a few isolated reports [85]. Another major issue is the need for dry conditions, meaning that after hybridization, the QCM must be dried for accurate measurements, due to the significant vibrational damping imposed by liquid medium. For most systems, liquid phase analysis is critical and the advantages of performing measurements in a liquid environment are obvious.

#### 6.4.2. Cantilever-based detection

Cantilever-based detection systems replaces the QCM with a miniature cantilever, typically fabricated from silicon or some other crystalline material, though there have been reports of polymer-based cantilevers. The cantilevers are oscillated, usually through piezoelectric means [157,158]. Conventional detection is performed by monitoring the deflection of laser light off the surface of the cantilever tip. Measurements of resonant frequency shifts due to hybridization of target DNA with immobilized single-stranded capture DNA has been achieved using these systems, and has proven to possess sufficient sensitivity to detect single-base mismatches, as well as differentiation between complementary and non-complementary sequences [159]. The presence of laser and signal detection instrumentation, however, poses a challenge in miniaturization.

## 7. Integrated Pathogen Detection Systems

Integration of all the microfabricated components needed to perform DNA detection to achieve portable, automated raw-sample-to-result functionality is no easy task. Several groups have already begun to address this challenge, with the incorporation of micropumps, microvalves, micromixers, heaters, detectors, and other analytical components. Significant progress has been demonstrated in various types of platforms including but not limited to, capillary driven test strips, centrifugal microfluidic devices, droplet-based microfluidic platforms, and large-scale system integration platforms. Though true μTAS systems are primarily still in the laboratory development stage, partially integrated μTAS devices have been developed for commercial applications. Of the different types of μTAS systems, PCR microfluidics is among the most prevalent and has been integrated with on-chip sample preparation and capillary electrophoresis.

**Figure 10.**
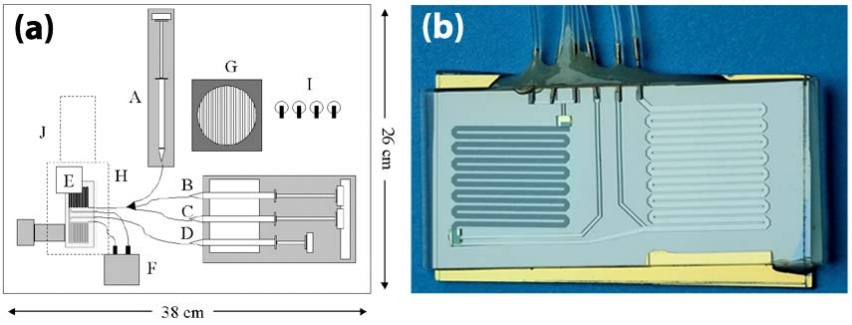
(a) A schematic of the assembled bacterial detection system is shown. The system includes integrated syringe pumps (A-D), Moog micro valve (F), cooling fan (G), LED-based fluorescence excitation / detection system (H – dotted outline) with PMT detector (J), power toggle switches (I). The microfluidic purification/detection chip (E) is inserted into the unit directly above the thermoelectric heater cooler. The syringes are connected to the chip via TygonTM tubing (black lines) and contain the sample lysate (A), ethanol wash buffer (B), dH_2_O (C), and PCR master mix (D). The Moog micro valve (F) is also connected to the chip via tubing and controls pressurization and fluid flow through the chip outputs. The entire unit measures 36cm × 28cm × 15cm. Reprinted with permission [32], ^©^2003 Elsevier Science B.V.; (b) Photographic image of the microfluidic purification/detection chip (E).

The functional integration of PCR and capillary electrophoresis on a single microchip was successfully integrated by Koh *et al.* for the detection and identification of two model bacteria, *Escherichia coli O157* and *Salmonella typhimurium* [160]. Similar systems were fabricated in a variety of different materials, namely PMMA, polycarbonate, and PDMS [15]. More recently, DNA purification and real-time PCR were successfully integrated for the single-chip detection of *Listeria monocytogenes* by Cady *et al.* on a portable instrument shown in Figure 10(a) with on-board pumping, valving, thermal cycling, and detection functionalities. The single-chip is shown in Figure 10(b).

In this work, DNA purification was performed by running lysed cell samples through a channel arrayed with 10 μm silicon oxide pillars and PCR was conducted in a serpentine amplification channel using an external thermal cycler, and real-time detection was performed with LED-excitation of TaqMan and measurements using a miniaturized PMT [32,48]. Automated sample preparation PDMS chips have also been developed to isolate nucleic acids from small numbers of bacterial cells with all cell isolation, cell lysis, DNA and mRNA purification and recovery processes carried out on a single standalone nanoliter-volume chip [161,162]. A fully-integrated chip for immunomagnetic bead-based sample preparation, PCR, and DNA microarray detection has been developed for the detection of *Eschericia coli* K12 from rabbit blood samples [163]. Other fully-integrated DNA-based assays that have been shown to be capable of multiple pathogen detection make use of individual electrode surfaces immobilized with capture probes [164,165]. In one particular system, signal amplification is achieved via tagging of the target DNA with gold nanoparticles and detection is based on measuring the amount of subsequent electrocatalytic deposition of silver metal onto the nanoparticles [165]. Other integration formats include the innovative compact disk device, also known as LabCD [166], a commercial product which utilizes centrifugal forces for pumping of fluids through reservoirs, valves, mixing chambers, and heating chambers. The control of flow rates through the device is tuned via different disk spin speeds, and is capable of achieving sample preparation, DNA purification, and PCR amplification.

In the laboratory setting, fully integrated systems exist for DNA analysis of complex biological samples employing the concept of raw-sample-to-result. One such system employs thermally-actuated paraffin-based microvalves and electrochemical and thermopneumatic pumps to achieve sample preparation (magnetic bead-based cell capture, cell pre-concentration and purification, and cell lysis), PCR, DNA hybridization and electrochemical detection on a single device. It has been demonstrated to show detection of pathogenic bacteria from milliliters of whole blood samples. An integrated portable genetic analysis system for pathogen detection has been prototyped by Mathies *et al.* using rapid PCR amplification followed by capillary electrophoretic separation of labeled analyte and fluorescent detection, and has been demonstrated directly on *E. coli* and *Staphylococcus aureus* cells [167]. In the push for detection in smaller sample volumes, fully integrated nanoliter-volume systems have also been developed in recent years [168]. The commercially-available Cepheid GeneXpert^®^(GX) system employs single-use microfluidic cartridges to integrate sample preparation, amplification, and detection. Utilized by the U.S. Postal Service for the detection of anthrax spores, the GX system has been shown to be both user-friendly and effective [169,170]. Other commercial μTAS systems for DNA analysis have also been developed by numerous microfluidic companies in a variety of formats and functionalities, including ACLARA BioSciences, Fluidigm Corporation, Affymetrix, Agilent Technologies, Alderon Biosciences, Roche Molecular Diagnostics, and Motorola Inc [15,171].

## 8. Conclusion and Future Directions

The development of a fast, sensitive, multiplexed, and easy to operate pathogen sensing systems will have global impacts on healthcare, agriculture, environmental monitoring, and bio-defense. Different strategies have been used in both research and commercial settings to develop nucleic acid-based sensors and lab-on-a-chip systems. With the need for portable, disposable DNA chips to replace traditional, expensive, and bulky instrumentation, applications for DNA-sensing devices are being rapidly driven towards rapid pathogen detection, DNA sequencing, and drug discovery. Miniaturization and reliability are the main challenges to widespread distribution of portable nucleic acid-based sensors with raw-sample-to-result functionality. One major bottleneck limiting the portability of nucleic acid-based microfluidic system lies in the difficulty of integrating sample preparation. In order to realize these handheld diagnostic systems, on-chip processing of raw samples and mastery of automated microfluidic control must be achieved. Enabling technologies discussed in this review will have a significant impact on the future development of handheld (point of care) nucleic acid-based detection systems. As mentioned in this review, dielectrophoretic (DEP) sample preparation, filtration-based separation, and immunomagnetic separation are all viable options for enriching target microorganisms from samples. Although DEP offers several advantages over other methods, such as its ability to distinguish between live and dead cells, this technology is more difficult to integrate into miniaturized systems. This is primarily due to complex electronic control architectures, and the incompatibility of this technique with heterogeneous sample matrices. At this time, filtration-based sample preparation, followed by immunomagnetic separation, is the most compatible purification technology with point of care systems. Passive methods, such as those offered by capillary forces, gravity, or creative topography are generally preferred due to lower power consumption, but battery-powered or hand-powered options can also provide a practical approach [34].

Following sample preparation, nucleic acid extraction/purification and nucleic acid detection must be addressed for the development of viable point of care detection systems. Multiple research groups have demonstrated that a microfluidic solid phase extraction approach towards nucleic extraction and purification is most compatible with miniaturized devices. The use of solid phase resins or microfabricated structures provides compatibility with microfluidic architectures and reduces the total volume of purified nucleic acid for subsequent detection. Detection technologies must also be optimized for point of care systems. As described, fluorescence-based detection methods, such as fluorogenic real-time PCR provide extremely high sensitivity. The complexity of the detection optics may limit the applicability towards miniaturized devices, but multiple research groups, including ours, have demonstrated optical detection in portable, low-power platforms. Competing methods, such as electrical detection may not provide the needed sensitivity for nucleic acid-based detection, but could offer less complex detection components. Of the currently used electrical techniques, impedance-based methods provide the highest sensitivity with the most information-rich output, and should be further investigated. Mechanical detection methods often require complex optical or electrical analysis (such as cantilever-based techniques), which make them no better suited to point of care applications than optical methods. From this standpoint, future point of care detection systems will mostly likely be based upon a microfluidic platform using solid phase extraction, PCR amplification, and a fluorescence-based optical readout. Concurrent development of high sensitivity electrical detection methods, such as transistor-based detection, may yield effective detection elements for far-term analytical systems.

To further decrease processing time and detection limits, further investigations into technologies with even higher specificity and sensitivity are needed. Nanotechnology will play a vital role in the development of new techniques for nucleic acid detection. The implementation μTAS systems will allow for easy standardization of methods necessary for the reliability and repeatability of results. However, the need for disposability imposes a limit on the size and cost of portable sensors, hence increasing the complexity of the technology may become too uneconomical for production. Much of the current published work, however, is promising, utilizing passive microfluidic components that are easily integrated into disposable devices. And, efforts to push towards non- or minimally instrumented diagnostic devices are in place [17].
